# Isolation, Identification, Genomic Analysis, and Rescue of a Field-Derived Isolate of Reticuloendotheliosis Virus from Hubei, China

**DOI:** 10.3390/vetsci13101084

**Published:** 2026-10-13

**Authors:** CE Chen, Mengjin Tian, Mei Zhang, Yuying Yang, Xiongyan Liang

**Affiliations:** College of Animal Science and Technology, Yangtze University, Jingzhou 434025, China; ccsj1224@outlook.com (C.C.);

**Keywords:** Gammaretrovirus, reticuloendotheliosis virus, virus isolation, virus identification, consensus genome sequence, genomic characterization, gp90 protein, phylogenetic analysis, infectious clone, virus rescue

## Abstract

Reticuloendotheliosis virus (REV) is an oncogenic retrovirus that threatens the global poultry industry through immunosuppression and neoplastic diseases. However, functional studies on field isolates have long been hampered by co-infections with other avian tumor viruses and complex genetic backgrounds. To address these issues, a field-derived REV strain, designated HB2301, was obtained from chickens in Hubei Province, China. These chickens exhibited emaciation, depression, and decreased egg production, and tumor-like nodules were observed in visceral organs at necropsy. The isolate was confirmed as REV-positive by multiplex PCR and IFA, with no detectable ALV (A/B/J/K) or MDV co-infection. Sequence analysis of the 8285 bp consensus proviral genome classified HB2301 into antigenic Subtype III. The REV genome proved highly conserved overall; the long terminal repeat (LTR) region exhibited greater variation than the coding regions, while the *gag* and *pol* genes remained the most conserved. Further analysis of the REV gp90 protein identified a unique valine-to-methionine point mutation at amino acid position 72 (V72M). This mutation is exclusively present in HB2301 and a previous Hubei isolate (HB2015021), suggesting it functions as a candidate regional marker rather than a random variation. Following consensus genome sequencing, a full-length infectious clone plasmid (pMD18-REV ABC) was constructed utilizing seamless cloning and enzymatic digestion, incorporating a targeted synonymous mutation (A2649C) as a specific molecular marker. Transfection of the infectious clone into DF-1 cells successfully rescued the recombinant virus rHB2301, which retained the A2649C genetic marker after three serial passages. The reactivity with an anti-REV gp90 monoclonal antibody and the in vitro replication kinetics of rHB2301 were comparable to those of the parental field-derived HB2301 stock. This study expands the epidemiological data on REV genetic evolution in China and provides a pure, genetically defined infectious clone platform that will facilitate future investigations into REV pathogenesis and support the development of novel prevention strategies.

## Simple Summary

Reticuloendotheliosis virus (REV) causes severe immunosuppression and tumor formation in poultry, presenting a continuous threat to the global poultry industry. In clinical settings, naturally occurring REV strains are often co-infected with other avian tumor viruses and carry complex genetic contamination, which significantly complicates efforts to study how the virus causes disease. To overcome this obstacle, researchers obtained a field-derived REV isolate from infected chickens and determined its consensus genome sequence. Using this genetic blueprint, they engineered a pure, artificially rescued version of the virus containing a specific genetic tracking marker. In vitro tests showed that the rescued virus had replication and antigenic reactivity comparable to the parental field-derived stock. This approach provides researchers with a clean, interference-free tool to study REV pathogenesis.

## 1. Introduction

Reticuloendotheliosis (RE) is an avian disease syndrome caused by infection with the reticuloendotheliosis virus (REV) and is characterized by immunosuppression, growth retardation, lymphoid hyperplasia, and tumor formation [1,2]. Since its initial isolation from turkeys in 1958, REV has disseminated globally across Asia, Europe, the Americas, and Africa, posing a persistent threat to the worldwide poultry industry [3,4,5,6,7]. As a single-stranded RNA virus belonging to the genus Gammaretrovirus, the REV proviral genome comprises typical structural (*gag*), enzymatic (*pol*), and envelope (*env*) genes flanked by long terminal repeats (LTRs) [8]. While all REV isolates share strong antigenic cross-reactivity and thus belong to a single overall serotype, minor yet distinct antigenic variations exist among them. These minor differences allow the single serotype to be further divided into three antigenic subtypes based on subtle variations in neutralization titers and differential reactivity with monoclonal antibodies [9]. 

As a retrovirus, the REV genome is highly susceptible to integration into the genomes of other viruses, such as fowlpox virus (FPV) and Marek’s disease virus (MDV); under certain conditions, infectious REV virions can be released during replication of these carrier viruses [10,11]. Currently, FPV strains carrying integrated REV genomic sequences have become predominant circulating strains in China, Australia, and the United States [12]. However, the length of the integrated REV sequences varies among strains, resulting in a complex viral genetic background [12,13]. Direct utilization of field-isolated wild-type strains for REV research presents distinct limitations: first, these strains are frequently associated with multi-pathogen co-infections (e.g., avian leukosis virus (ALV) and MDV) and genomic integration events [14,15,16,17]. Second, they preclude the execution of precise genetic point mutations or recombinant modifications. 

To overcome these limitations, as a core tool of reverse genetics, infectious clone technology enables the stable preservation and artificial rescue of the entire viral genome at the plasmid DNA level. This provides an important platform for strictly eliminating contaminant interference and deeply elucidating viral gene functions and pathogenic determinants [18]. In this study, a field-derived REV isolate (HB2301) was isolated from chickens exhibiting neoplastic disease at a local farm in Hubei Province. Following sequencing and assembly of the consensus proviral genome, a full-length infectious clone plasmid was successfully constructed, and the biologically active recombinant virus rHB2301 was rescued. The consensus genome of the field isolate obtained herein enriches the REV genomic database in China, providing primary data for monitoring the mutations of circulating strains. Furthermore, the rescued recombinant virus, characterized by a clear genetic background and high purity, reduces interference from detectable co-infecting field pathogens. This establishes a crucial material foundation for future in-depth investigations into the molecular pathogenic mechanisms of key REV proteins and their host interaction networks.

## 2. Materials and Methods

### 2.1. Clinical Examination, Cells and Antibodies

In October 2023, chickens at a commercial native chicken farm in Hubei Province, China, exhibited clinical symptoms including emaciation, depression, and a decline in egg production. According to the farm veterinarian, the morbidity and mortality rates of the flock reached 7.6% and 5.5%, respectively. The carcasses of two 17-week-old diseased chickens presenting with obvious clinical signs were submitted to our laboratory for further diagnosis. DF-1 cells were purchased from Pricella Biotechnology (Wuhan, China). The mouse anti-REV gp90 monoclonal antibody (10A 21) was preserved by our laboratory.

### 2.2. Virus Isolation, Culture, and Identification

In a biosafety cabinet, 100 mg of spleen and liver tissues from the diseased chickens were placed into a 2 mL centrifuge tube, mixed with 0.9 mL sterile PBS and steel beads were added, homogenized at low temperature, and then centrifuged at 4 °C and 12,000 r/min for 5 min. The supernatant was collected for subsequent virus inoculation. The pellet was collected for tissue DNA extraction using a Tissue/Cell DNA Extraction Kit (Aidlab, Beijing, China) according to the manufacturer’s instructions. The samples were initially screened for REV, ALV, and MDV by multiplex PCR with specific primers (Table 1). The ALV, MDV and REV cases used as positive controls in PCR were archived in our laboratory [19]. PCR-positive homogenates were then subjected to three freeze–thaw cycles, centrifuged at 12,000 r/min for 10 min, and the resulting supernatant was inoculated into DF-1 cells at a 1:10 volume ratio. After adsorption at 37 °C for 1 h, the cells were incubated at 37 °C in a 5% CO_2_ atmosphere, with uninoculated DF-1 cells maintained as a negative control, and the virus was blind-passaged for five generations.

To confirm viral infection, fifth-passage cells grown in 24-well plates to 60–70% confluence were processed for indirect immunofluorescence assay (IFA). Briefly, cells were fixed with 4% paraformaldehyde (15 min), permeabilized with 0.5% Triton X-100 (15 min), and blocked with PBS containing 10% FBS (SORFA, Beijing, China) for 1 h at 37 °C. They were then incubated with the mouse anti-REV gp90 monoclonal antibody (10A 21) for 1 h, followed by a 45-min incubation in the dark with FITC-labeled goat anti-mouse IgG (Proteintech Group, Wuhan, China). After thorough PBS washes between each step, fluorescence was visualized under an inverted fluorescence microscope (Ningbo Sunny Instruments Co., Ltd., Zhejiang, China).

The REV-positive isolate was subsequently titrated by inoculating 10-fold serial dilutions (10^−1^ to 10^−10^) into 80% confluent DF-1 cells in 96-well plates, with eight replicates per dilution and negative and blank controls. Following a 1 h adsorption period at 37 °C, the inoculum was replaced with DMEM supplemented with 2% FBS. After 7 days of culture, the cells were fixed and stained using the same IFA protocol described above. The number of IFA-positive wells was recorded, and the viral titer was calculated as TCID_50_ per 100 μL by the Reed–Muench method.

### 2.3. Viral Genome Amplification and Sequence Analysis

Using the DNA extracted in Section 2.2 as a template, and based on the published DNA sequence of the REV-HLJR0901 strain (GenBank accession number GQ415646.2), six pairs of primers were designed using Primer Premier 5.0 software (Premier Biosoft, San Francisco, CA, USA) for whole-genome PCR amplification (Table 2). All oligonucleotide primers were synthesized by Wuhan Hecegene Technology Co., Ltd., Wuhan, China. PCR products matching the expected size were sent to Wuhan Hecegene Technology Co., Ltd. for sequencing. The overlapping sequences were assembled using the SeqMan function within the DNASTAR Lasergene v7.1 sequence analysis software (DNASTAR, Madison, WI, USA) to obtain the consensus sequence of the proviral genome. 

Reference strains (Table S1) were downloaded from GenBank [17,20,21,22,23]. The complete genome of the isolate was aligned with published REV strains using the Clustal W alignment method within the Sequence Demarcation Tool (SDT) (v. SDTv1.3) software to conduct molecular evolutionary genetics analysis. A phylogenetic tree was constructed using MEGA 12.0 and subsequently optimized using iTOL (https://itol.embl.de/tree/) (accessed on 7 July 2026).

### 2.4. Virus Rescue

**Figure 1:**
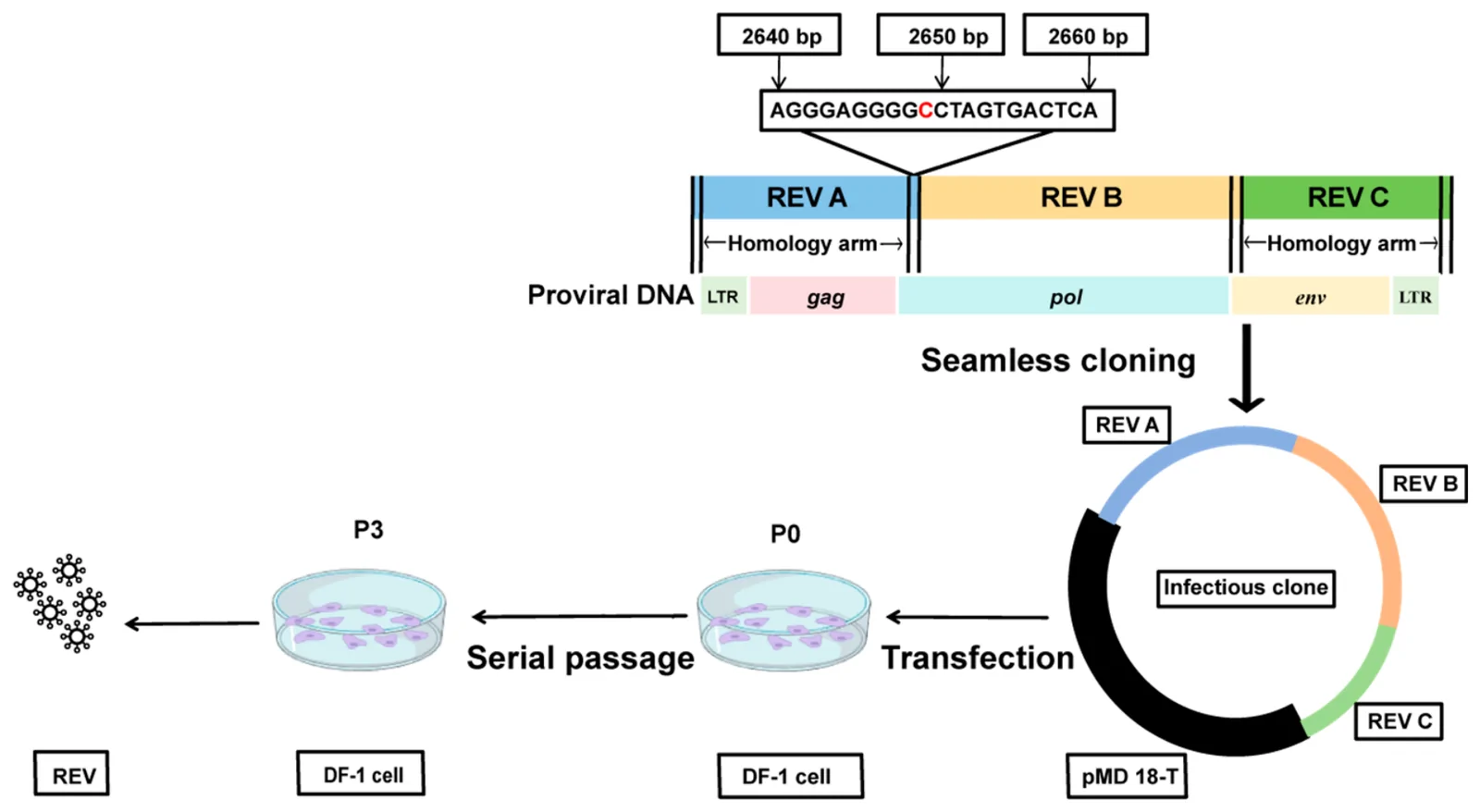
Schematic diagram of the construction of the infectious clone plasmid and the virus rescue strategy.

The proviral genome was divided into three overlapping fragments: REV A, REV B, and REV C (with a 35 bp overlap between A and B, and a 15 bp overlap between B and C). Vector homology arms were attached to the 5′ end of REV A, the 3′ end of REV B, and the 3′ end of REV C. Fragment-overlap homology arms were introduced at the REV A–REV B and REV B–REV C junctions by the corresponding adjacent primers (Table 3). Each 25 µL PCR reaction contained 12.5 µL of 2× A8 FastHiFi PCR MasterMix (Aidlab, Beijing, China), 1 µL each of the forward and reverse primers, 1 µL of template, and ddH_2_O to 25 µL. The thermal cycling conditions were 95 °C for 5 min, followed by 31 cycles of 95 °C for 20 s, 52 °C for 20 s, and 72 °C for 1 min, with a final extension at 72 °C for 5 min. For seamless assembly, the vector and insert fragments were mixed at a molar ratio of 1:3 and incubated at 50 °C for 30 min. REV A and REV B were inserted into the pMD18-T vector via homologous recombination using the Seamless Assembly Cloning Kit (CloneSmarter Technologies Inc., Houston, TX, USA) to construct the pMD18-AB plasmid. The pMD18-AB plasmid was then linearized by double digestion with *Afl*II and *Eco*RI in a 50 µL reaction containing 1 µg DNA, 1 µL each enzyme, and 1× rCutSmart Buffer at 37 °C for 5 h. After purification, REV C was inserted into the linearized vector by seamless assembly to generate the full-length infectious clone plasmid pMD18-ABC. A synonymous mutation (A→C) was introduced at genome position 2649 bp using primers REV-A R and REV-B F to eliminate a *Spe*I restriction site, serving as a genetic marker (Figure 1). Following construction, the infectious clone plasmid was sent to Wuhan Hecegene Technology Co., Ltd. for sequencing verification. The synonymous nature of the A2649C mutation was further confirmed by the alignment of nucleotide and amino acid sequences (Figure S3).

The verified recombinant plasmid pMD18-REV ABC was transfected into DF-1 cells using the TurboFect Transfection Reagent (Thermo Fisher, Beijing, China) and incubated at 37 °C in a 5% CO_2_ atmosphere. 

### 2.5. Evaluation of the Virus Rescue

Once the cells reached confluence, they were blind-passaged for three generations. To further verify viral rescue and rule out potential artifacts such as residual plasmid DNA, direct proviral expression, or cell transfer, the third-passage transfected cell cultures were harvested, subjected to three freeze–thaw cycles to release intracellular virions, clarified by centrifugation at 12,000 rpm for 10 min, and filtered through a 0.22 μm membrane to obtain clarified, cell-free supernatant. To completely remove free plasmid DNA, the supernatant was treated with DNase I prior to inoculating fresh DF-1 cells, and subsequent passages were strictly performed using this clarified, DNase-treated, cell-free supernatant. Following serial cell-free passages, viral replication was confirmed via an IFA utilizing the mouse anti-REV gp90 monoclonal antibody 10A 21 as the primary antibody. Meanwhile, cellular RNA was extracted and treated with gDNA Remover to eliminate genomic/plasmid DNA contamination before reverse transcription. RT-PCR targeting the REV LTR region was performed with parallel no-RT, mock-infected, and plasmid backbone controls, and the total cDNA obtained by reverse transcription was submitted to Wuhan Hecegene Technology Co., Ltd. for sequencing to confirm the presence and stability of the engineered A2649C marker.

The TCID_50_ of the rescued virus was determined following the procedure described in Section 2.2. To evaluate viral growth kinetics, DF-1 cells were seeded into 6-well plates at a density of 5 × 10^5^ cells/well and cultured overnight. The cells were subsequently inoculated with either the parental field-derived HB2301 stock and the recombinant virus rHB2301 at an identical multiplicity of infection (MOI), utilizing three replicates per virus. Following a 1 h adsorption period, the cells were washed three times with PBS and maintained in DMEM supplemented with 2% FBS. Viral supernatants were collected every 24 h for 7 consecutive days; at each time point, 200 µL of supernatant was collected from each well, clarified by centrifugation at 3000 rpm for 10 min, and replaced with an equal volume of fresh DMEM supplemented with 2% FBS to maintain a constant culture volume, with the maintenance medium not fully replaced. Viral titers (TCID_50_) were determined as previously described. The in vitro proliferation curves were ultimately generated using GraphPad Prism 6.0 software.

## 3. Results

### 3.1. Isolation and Identification of the REV Strain

**Figure 2:**
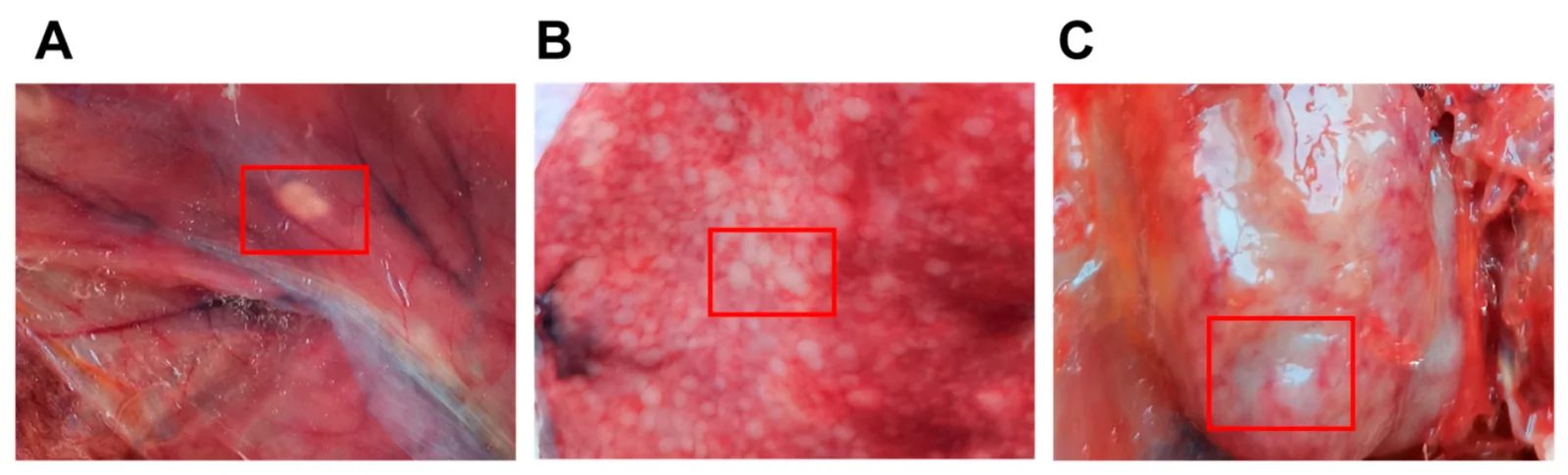
Gross necropsy findings in the diseased chickens. (**A**) Raised white nodular lesion on the visceral peritoneum of chicken No. 1. The lesion is indicated by a red box. (**B**) Tumor-like nodules in the liver of chicken No. 1. The lesion is indicated by a red box. (**C**) Tumor-like nodules in the kidney of chicken No. 2. The lesion is indicated by a red box.

Postmortem examination revealed gross tumor-like nodules in the diseased chickens. White tumor nodules were observed on the visceral peritoneum (Figure 2A). Severe pathological changes were particularly evident in the visceral organs, notably the liver and kidneys. The liver was congested and swollen with a tense capsule and blunt margins, and its surface and cut sections were densely covered with numerous white tumor-like nodules (Figure 2B). Both kidneys were markedly enlarged and diffusely infiltrated by abundant grayish-white neoplastic nodules; in localized areas, the normal renal parenchyma was completely replaced by tumor tissue (Figure 2C).

**Figure 3:**
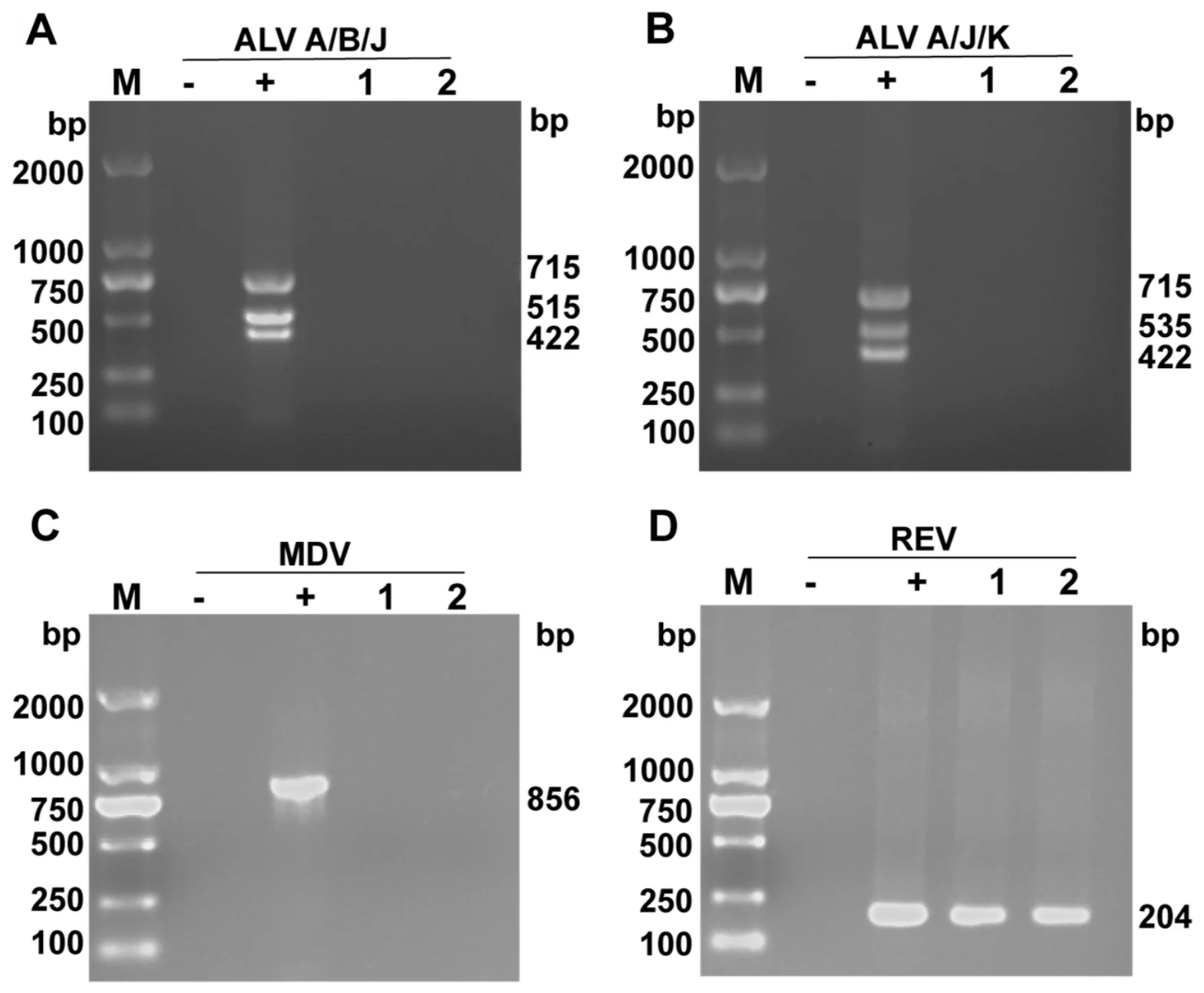
PCR detection results. (**A**) Multiplex PCR amplification of ALV subgroups A, B, and J. (**B**) Multiplex PCR amplification of ALV subgroups A, J, and K. (**C**) PCR amplification of MDV. (**D**) PCR amplification of REV. Note: Lane M: DNA Marker AL2000 (Aidlab, Beijing, China); Lane −: negative control; Lane +: positive control; Lanes 1 and 2: clinical samples.

The Multiplex PCR results showed that no target bands were amplified for any of the ALV subtypes (A, B, J, K) or MDV (Figure 3A–C). Only a specific DNA band of approximately 204 bp, consistent with the expected size of the REV target gene, was successfully amplified from the diseased chicken tissues (Figure 3D). The positive detection of REV by Multi-PCR indicates the presence of REV nucleic acid in the tissues of the diseased chickens.

**Figure 4:**
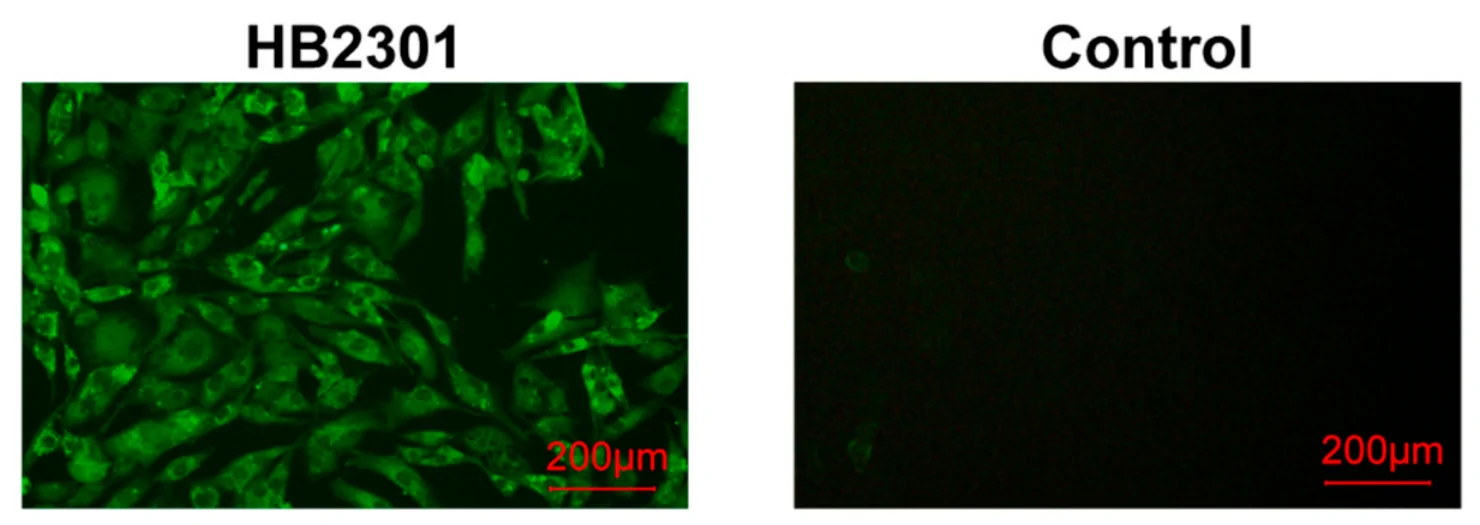
IFA identification results. Scale bar = 200 µm.

Following the inoculation of the processed tissue homogenates into DF-1 cells, the IFA revealed bright green specific fluorescence in the inoculated cells, whereas the uninoculated control cells exhibited no fluorescence (Figure 4). These results confirmed the successful isolation of an infectious REV, which was subsequently designated as the HB2301 isolate. TCID_50_ of the HB2301 isolate was determined using the Reed–Muench method, revealing a viral titer of 10^4.2^/100 μL for REV HB2301.

### 3.2. Isolation and Identification of the REV HB2301 Strain

**Figure 5:**
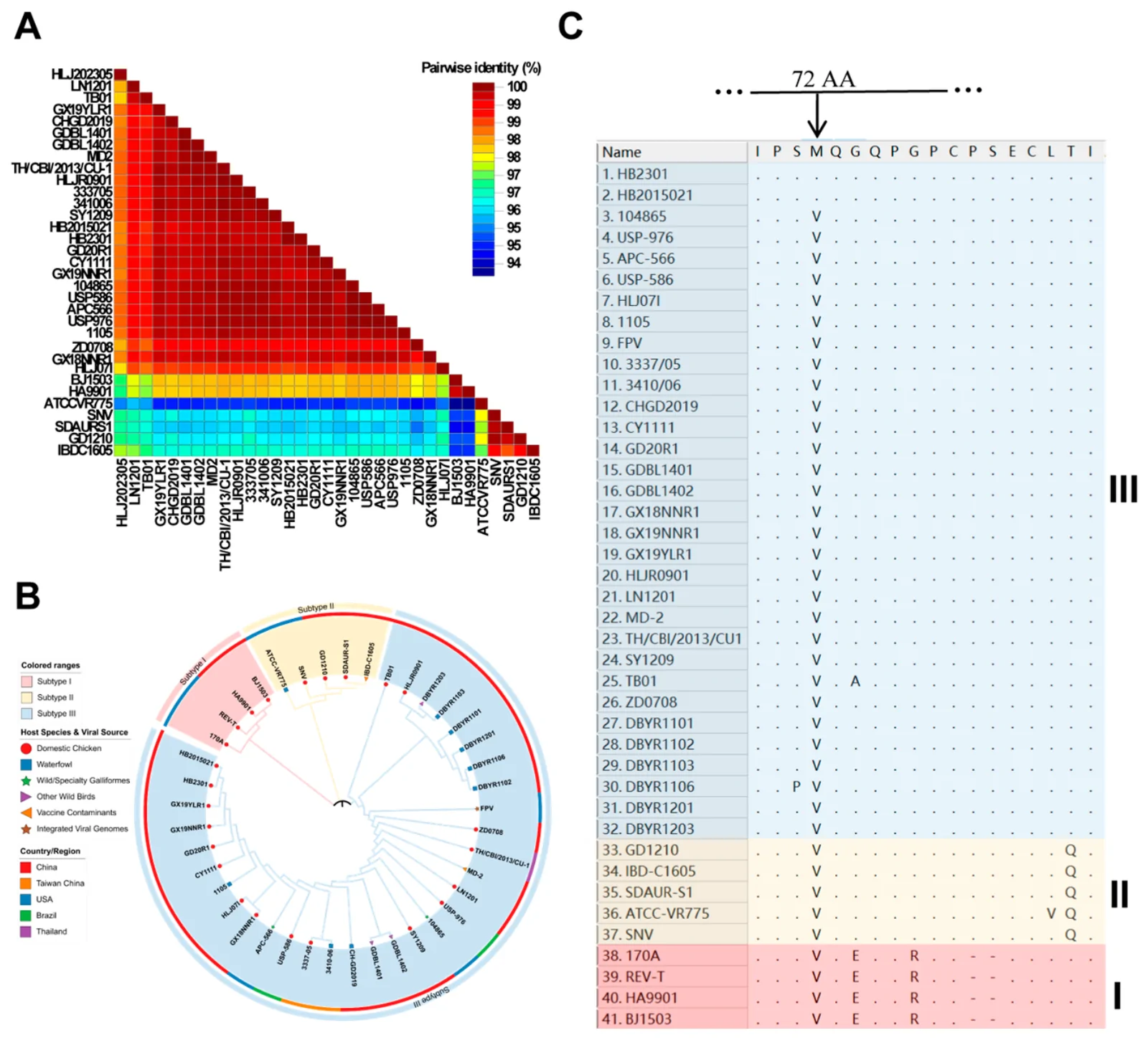
Comparative genomic and protein analysis of REV isolate HB2301 and reference strains. (**A**) Pairwise identity heatmap of full-length genome sequences, generated using ClustalW alignment and visualized with the Sequence Demarcation Tool (SDT) software. (**B**) Phylogenetic tree based on REV gp90 protein sequences, delineating the three subtypes (I–III) and incorporating host and geographic information. (**C**) Amino acid sequence alignment of partial gp90 protein. Subtype-specific conserved amino acid residues are highlighted across Subtypes I–III, including the unique V72M point substitution shared exclusively by Hubei isolates HB2301 and HB2015021.

To elucidate the genomic characteristics and phylogenetic relationships of the REV HB2301 strain, six pairs of specific primers were used to amplify the proviral genome of this strain in segments (Figure S1). The sequences were assembled using SeqMan to obtain the consensus full-length proviral genome sequence of 8285 bp (GenBank accession number: PZ879108). The genomic structure conforms to the characteristics of a replication-competent retrovirus, consisting of the 5′ LTR, *gag*, *pol*, *env*, and 3′ LTR (Table S2). Comparative alignment covering the complete 8285 bp genome generated a sequence identity matrix (Figure S2A). Strain HB2301 shared 97.9% nucleotide identity with Subtype I strains, 94.9–96.1% with Subtype II strains, and 98.4–100.0% with Subtype III strains. Whole-genome homology heatmap analysis (Figure 5A) indicated that REV genomes are highly conserved overall. Although minor variations were present among lineages, strain HB2301—positioned within the predominant epidemic cluster—maintained over 94.9% pairwise identity with ATCC-VR775, the most divergent reference strain.

To clarify subtype classification, a phylogenetic tree based on the REV gp90 protein was constructed (Figure 5B). Reference strains were segregated into three independent evolutionary clades (Subtypes I–III): Subtype I: Comprised classic reference strain REV-T and early domestic isolates, including HA9901, BJ1503, and 170A; Subtype II: Consisted of ATCC-VR775, SNV, GD1210, SDAUR-S1, and vaccine contaminant strain IBD-C1605; Subtype III: Formed the largest clade, encompassing recent domestic and international clinical isolates as well as wild bird/waterfowl-origin strains. Strain HB2301 clustered within Subtype III and localized to the tip of a minor sublineage alongside regional strain HB2015021 (2015), indicating a close ancestral relationship.

Segmental genomic analysis demonstrated that nucleotide divergence within LTR regions was higher than in coding regions across subtypes. The LTR sequence of HB2301 exhibited high homology (99.6–100.0%) with most Subtype III strains (HB2015021, USP-976, GD20R1, CU-1), showing evolutionary divergence of 0.0–0.4%. Identical LTR comparisons yielded 96.8–97.1% homology with Subtype I strains (HA9901, BJ1503) and 91.3–96.7% with Subtype II strains (GD1210, SNV, SDAUR-S1, ATCC-VR775) (Figure S2B). Genes encoding structural and enzymatic proteins (*gag* and *pol*) remained the most conserved across the genome (Figure S2C,D). Within Subtype III, intra-lineage identity exceeded 99.5%, while comparisons against Subtype II reference strains maintained 95.9–96.9% identity.

Amino acid alignment of the gp90 protein revealed lineage-specific conserved substitutions alongside sporadic intra-subtype mutations (Figure 5C). Subtypes I and II contained fixed mutation profiles (e.g., E/R substitutions in Subtype I; Q substitution in Subtype II). Conversely, strains HB2301 and HB2015021 displayed a unique point mutation at position 72 of the gp90 protein, where valine was substituted by methionine (V72M). Because this alteration occurred exclusively in these two Hubei isolates, V72M serves as a candidate regional marker rather than a stochastic variation.

### 3.3. Construction of the REV Infectious Clone Plasmid

**Figure 6:**
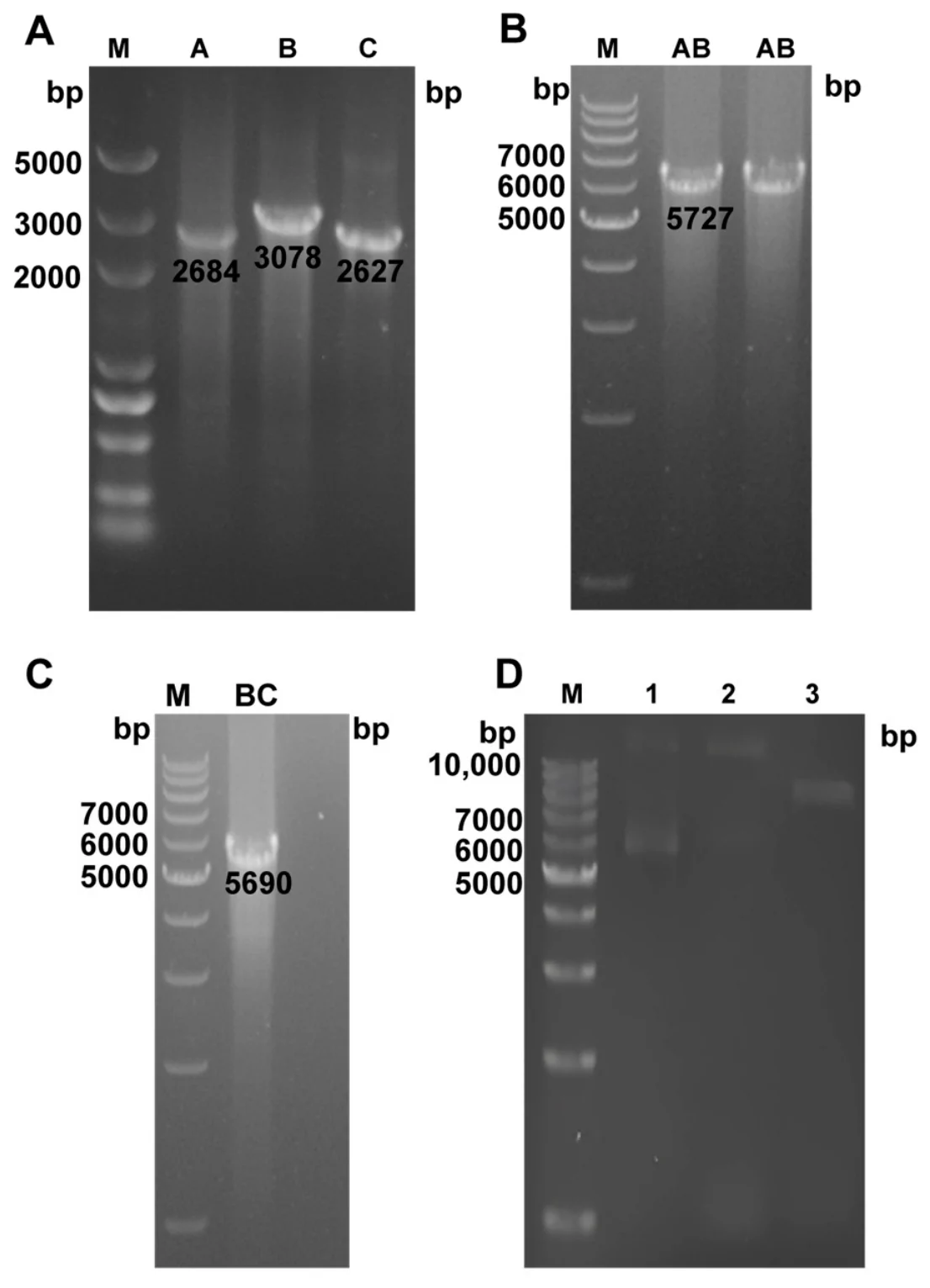
Construction and verification of the REV HB2301 infectious clone. (**A**) PCR amplification of three overlapping target fragments A, B, and C using the proviral DNA of the HB2301 strain as a template. (**B**) PCR identification of the intermediate plasmid pMD18-REV AB. The amplified fragment AB confirms the successful ligation of fragments A and B. (**C**) PCR identification of the full-length recombinant plasmid pMD18-REV ABC. The presence of the REV BC target fragment (5690 bp) indicates the correct insertion of the full-length genome into the pMD18-T vector. (**D**) Results of double restriction enzyme digestion of pMD18-REV ABC plasmid. Lane M: 1kb DNA Ladder Marker (Aidlab, Beijing, China); Lane 1: pMD18-REV ABC plasmid; Lane 2: *Spe*I single digestion of the pMD18-REV ABC plasmid Lane 3: Double digestion product of the pMD18-REV ABC plasmid.

To construct the infectious clone plasmid for REV HB2301, the proviral DNA of the strain was used as a template to amplify three overlapping target fragments: A (2684 bp), B (3078 bp), and C (2627 bp) (Figure 6A). Employing a stepwise cloning strategy, fragments A and B were first assembled to generate pMD18-REV AB, which was confirmed as a positive clone via PCR (Figure 6B). Subsequently, fragment C was inserted to yield the recombinant plasmid pMD18-REV ABC, which contained the full-length REV proviral genome. PCR verification confirmed the presence of the target REV BC fragment, indicating the correct insertion of the full-length genome into the pMD18-T vector (Figure 6C). Further double digestion of pMD18-REV ABC with *Afl*II and *Spe*I produced only a single linearized band (Figure 6D), demonstrating that the original *Spe*I restriction site in the REV HB2301 genome had been effectively abolished. These results confirmed the successful construction of the infectious clone plasmid, incorporating a genetic marker for subsequent virus rescue.

### 3.4. Verification of the Rescued Virus

**Figure 7:**
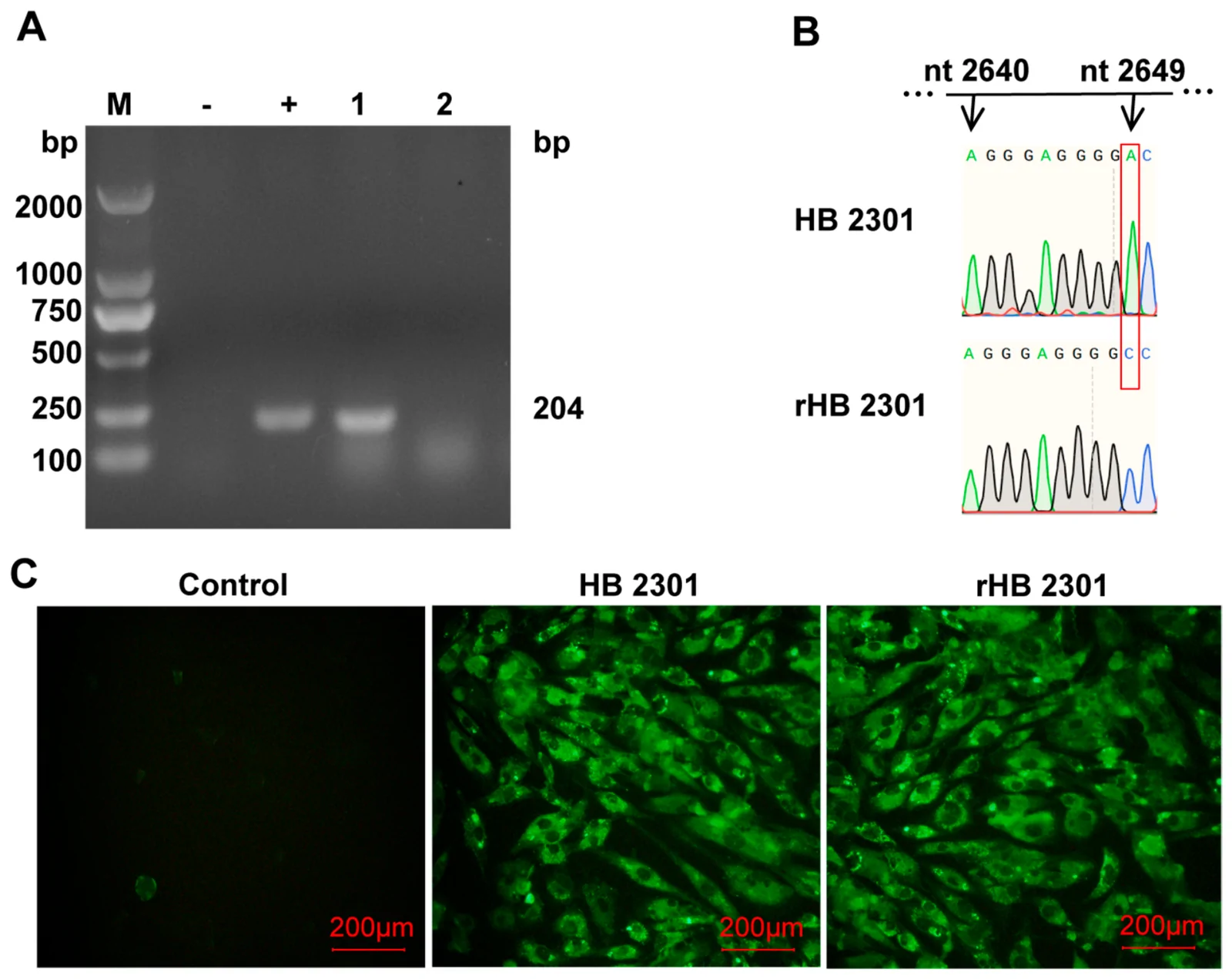
Verification of virus rescue and biological characterization of the recombinant virus rHB2301. (**A**) RT-PCR detection of REV LTR from DF-1 cells inoculated with DNase-treated, cell-free supernatant after serial passages. Lane M: DNA Marker AL2000; Lane −: Negative control; Lane +: positive control; Lane 1: Samples; Lane 2: No-RT control. (**B**) Sequencing analysis of the viral cDNA. The chromatogram confirms the synonymous mutation from A to C at genomic position 2649 (highlighted by the red box) in the rescued virus (rHB2301) compared to the parental field-derived HB2301 stock. Numbers above the chromatograms indicate nucleotide positions (nt). (**C**) Indirect immunofluorescence assay (IFA) of DF-1 cells. Specific green fluorescence was detected in cells infected with both the parental HB2301 stock and the rescued virus rHB2301, whereas the mock-infected negative control exhibited no fluorescence. Scale bar = 200 µm.

To verify the virus rescue activity of the infectious clone and the biological characteristics of the rescued virus, pMD18-REV ABC was transfected into DF-1 cells and blind-passaged for three passages. To rule out potential artifacts such as residual plasmid DNA, direct proviral expression, or cell carry-over, the third-passage transfected cells were harvested, subjected to three freeze–thaw cycles, clarified by centrifugation, filtered through a 0.22-μm membrane, and treated with DNase I to obtain cell-free supernatant. This supernatant was then used to inoculate fresh DF-1 cells, and subsequent passages were strictly performed using the clarified, DNase-treated, cell-free supernatant. After serial cell-free passages, cellular RNA was extracted and treated with gDNA Remover before reverse transcription. RT-PCR targeting the REV LTR region was performed with parallel no-RT. The expected 204 bp LTR band was amplified from the inoculated DF-1 cells (Figure 7A), demonstrating that the rescued virus was infectious and able to establish a productive infection in fresh DF-1 cells.

Sequencing analysis of the viral cDNA confirmed an A-to-C mutation at nucleotide position 2649 of the genome (Figure 7B). This genetic marker was retained after three serial passages in the rescued virus, demonstrating that the recombinant virus was indeed derived from the constructed infectious clone. Furthermore, the indirect immunofluorescence assay (IFA) revealed that DF-1 cells infected with either the wild-type REV HB2301 strain or the rescued virus exhibited specific green fluorescence, whereas the negative control showed no fluorescence (Figure 7C). This indicates that the rescued virus can efficiently express viral proteins and showed reactivity with an anti-REV gp90 monoclonal antibody similar to that of the parental field-derived HB2301 stock.

**Figure 8:**
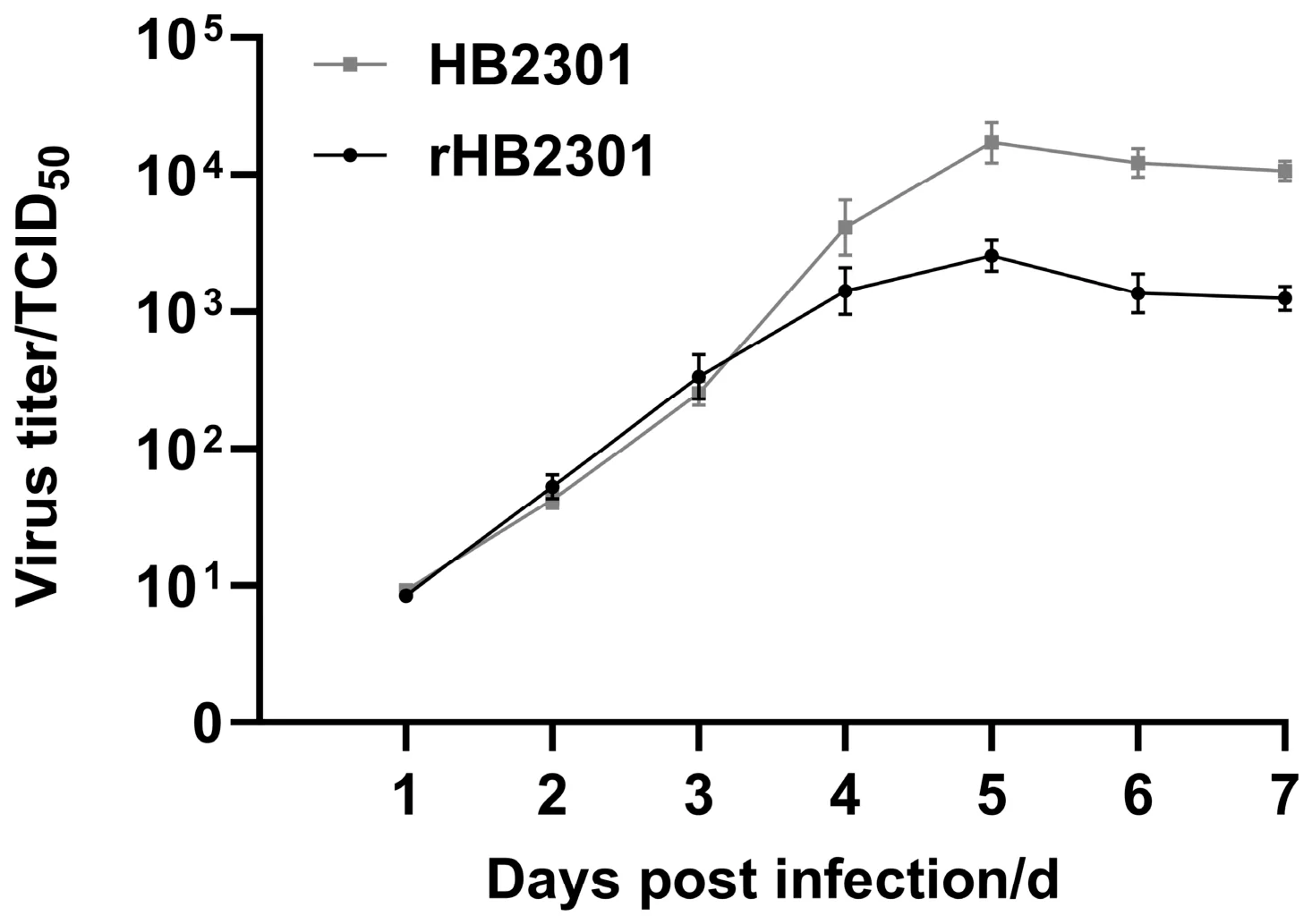
Kinetic curves of virus replication in vitro. Data were the mean ± SE (*n* = 3).

Further biological characterization revealed that the rescued virus, rHB2301, exhibited a TCID_50_ of 10^3.4^/100 μL and possessed infectious activity. In vitro replication kinetics demonstrated that rHB2301 showed a replication trend comparable to that of the parental field-derived HB2301 stock in DF-1 cells. Viral titers for both viruses peaked at 5 days post-infection (dpi). Across three independent biological replicates, the two viruses exhibited comparable replication kinetics with similar TCID_50_ values across all sampled time points (Figure 8). Taken together, the recombinant virus rHB2301, carrying a stable genetic marker and retaining infectious activity, was successfully rescued.

## 4. Discussion

In recent years, co-infections involving immunosuppressive pathogens have become highly prevalent in chicken flocks across China, with REV frequently forming severe co-infections alongside MDV and ALV [24]. REV not only independently induces immunosuppression and reticuloendotheliosis, but its genome also exhibits a notable propensity for integration into the genomes of large DNA viruses—such as FPV and pigeonpox virus (PPV)—and co-transmitting throughout host populations via these viral vectors [12,17]. Studies have confirmed that integrated REV sequences can be transcribed and released as infectious free REV virions in tissues supporting poxvirus replication, such as the chorioallantoic membranes (CAMs) of poxvirus-infected embryonated eggs, potentially exacerbating clinical immunosuppression and anemia [25]. Under such complex conditions of mixed infection and genomic integration, directly using clinical wild-type strains to investigate pathogenic mechanisms frequently introduces uncertainties due to interference from secondary pathogens or integrated sequences. To overcome these limitations, this study obtained and characterized a field-derived REV isolate, designated HB2301, from a local native chicken farm in Hubei Province, where postmortem examination revealed gross tumor-like nodules in the diseased chickens. Using homologous recombination combined with a restriction digestion strategy, we constructed the full-length infectious clone plasmid pMD18-REV ABC carrying a synonymous mutation (A2649C) as a genetic marker, and successfully rescued the recombinant virus rHB2301 with a pure genetic background in DF-1 cells.

Since REV was first isolated from turkeys in 1958, its prototype strain, REV-T, was identified as subtype I [26], with subsequent studies confirming its ability to induce reticuloendotheliosis across various poultry species, including chickens and quails [27]. To date, REV has been successfully isolated from a broad range of farmed and economically important avian species, including mallard [28], muscovy ducks [29] and domestic chickens [30]. Furthermore, multiple subtype III REV strains displaying high sequence homology have been detected in wild bird populations in northeastern China [30]. Internationally, REV has also been detected in mallards in Brazil [20], the endangered Attwater’s prairie chicken in the United States [22], and endangered endemic wild geese and albatrosses in Hawaii [31]. This broad host spectrum indicates that REV lacks strict host species barriers in nature. Wild waterfowl and migratory birds likely serve as natural reservoirs, facilitating inter-regional transmission and cross-species spillover into domestic poultry populations—a potentially important ecological driver underlying its widespread dissemination.

In this study, the host of the isolated HB2301 strain was a local Hubei chicken breed with an extended rearing cycle. Sequence analysis revealed that HB2301 shared the highest homology with HB2015021, an REV strain isolated in the same region in 2015, with both belonging to subtype III. This indicates the sustained persistence of subtype III strains within the local poultry population. Notably, HB2301 and HB2015021 harbor unique point mutations in the gp90 protein region that distinguish them from strains in other regions and subtypes. These regional mutations may reflect an adaptive evolution driven by viral adaptation to specific cellular receptors or immune pressures unique to local chicken breeds, offering a potential molecular target for identifying Hubei-endemic strains. Furthermore, subtype III strains isolated from wild birds in northeastern China (e.g., DBYR1203 and DBYR1101) and an isolate recovered from an MDV vaccine (MD-2) shared over 99.6% full-genome nucleotide identity with HB2301. They also exhibited 99.7% homology in the *env* gene with REV sequences integrated into FWPV, highlighting a high degree of cross-regional and cross-host genetic homogeneity within subtype III. Given Hubei’s dense river networks and the semi-free-range farming system prevalent for local chickens, these findings strongly support continuous cross-host transmission and regional epidemics of REV subtype III across the wild bird–waterfowl–poultry interface.

Further genomic analysis showed that the coding regions of REV—particularly the *gag* and *pol* genes—are highly conserved overall. This intrinsic genetic uniformity underscores the practical utility and value of the reverse genetics system established in this study. Because the rHB2301 infectious clone retains the authentic genomic backbone of contemporary subtype III wild strains (the primary circulating lineage), it serves not merely as an isolated Hubei strain, but as a functional molecular prototype for current circulating REV populations. Consequently, core viral characterizations performed using this defined genetic platform—such as assessments of polymerase activity, protein processing, or neutralizing antibody responses—are expected to offer insights that may be broadly applicable across diverse temporal and geographical REV isolates. Nevertheless, given the lineage-specific polymorphisms present in the LTR and the gp90 region of *env*, chimeric viruses constructed via fragment substitution may still be required to dissect strain-specific pathogenic variations, such as differences in tissue tropism or immunosuppressive potency—further highlighting the potential of this platform as a modular genetic tool. The rescued rHB2301 showed reactivity with an anti-REV gp90 monoclonal antibody and replication kinetics comparable to those of the parental field-derived HB2301 stock, with both reaching peak replication on day 5 post-infection, indicating efficient adaptability to standard laboratory cell lines. Throughout continuous serial passages, the engineered synonymous mutation marker (A2649C) was retained after three serial passages, demonstrating the genetic stability of this reverse genetics system during long-term experimental passage.

In summary, a reverse genetics system for HB2301, a currently circulating field-derived Subtype III REV isolate, was successfully established in this study. This system provides a pure and genetically well-defined molecular backbone representative of the REV population currently prevalent in China, serving as a valuable tool for dissecting viral pathogenesis and evaluating neutralizing antibodies. However, this study has two major limitations. First, the consensus genome was assembled from overlapping PCR amplicons and the infectious clone was derived from a single sequenced plasmid clone; therefore, these data cannot fully establish the genetic homogeneity of the original field stock or exclude the possibility of PCR-assembled chimeras. Second, validation was restricted to in vitro cell models, without in vivo pathogenicity experiments. Future studies will focus on characterizing the in vivo properties of rHB2301 in specific-pathogen-free (SPF) chickens to systematically elucidate its pathogenic spectrum, tissue tropism, and immunosuppressive capacity.

## 5. Conclusions

In conclusion, a field-derived Subtype III REV isolate, designated HB2301, was successfully isolated and characterized from diseased chickens in Hubei, China. Sequence analysis revealed a unique V72M substitution in the gp90 protein, which serves as a candidate regional marker. Furthermore, a highly stable reverse genetics system was constructed via seamless assembly, yielding a genetically pure recombinant virus (rHB2301) bearing an engineered synonymous marker. In vitro biological characterization demonstrated that rHB2301 replicated efficiently, retained the A2649C marker after three serial passages, and showed reactivity with an anti-REV gp90 monoclonal antibody comparable to that of the parental field-derived HB2301 stock. The establishment of this well-defined, interference-free reverse genetics platform provides a robust tool for future REV research. By employing site-directed mutagenesis or target gene deletions (e.g., targeting the LTR regulatory region or the *env* gene) in specific-pathogen-free (SPF) chicken models, this platform will facilitate systematic investigations into REV pathogenesis, tumorigenesis, and host immunosuppressive mechanisms. Moreover, the molecularly cloned rHB2301 recombinant virus offers a reliable model to elucidate potential synergistic co-pathogenesis and genomic integration between REV and other major avian pathogens (such as MDV, FWPV, and PPV), ultimately contributing to the rational design of targeted control strategies for poultry health.

## Figures and Tables

**Table 1:** Primers used for detection of ALV, MDV and REV.

Target Virus	Primer Sequence (5′ → 3′)	Length (bp)
REV-LTR	F: TGAGGGAAAATGTCATGCAACATCC	204
	R: ATCCCTACCCCACCCAGTAG	
ALV A	A/J/B/K F: CGGAGAAGACACCCTTGCT	
	A R: GCATTGCCACAGCGGTACTG	715
ALV J	J R: CGAACCAAAGGTAACACACG	422
ALV K	K R: TTGCGGCCTGGACCAATC	535
ALV B	B R: GTAGACACCAGCCGGACTATC	515
MDV	F: CTGACGGCCTATCTGAGGAG	856
	R: GGAAACCACCAGACCGTAGAC	

**Table 2:** Primers used for proviral genome amplification of REV.

Fragment	Primer Sequence (5′ → 3′)	Length (bp)
REV-F1	F: AATGTGGGAGGGAGCTC	952
	R: TCGATCCTGCCTGTCCCAT	
REV-F2	F: AAGAATGGACTGTCTCACC	1782
	R: ATTCTGGTCAAATCATTG	
REV-F3	F: GCTTGCGAATAATACTTTGGAG	2179
	R: CACAAGACGCCCTTCAGACT	
REV-F4	F: TGGAAGAAGAACTGTCCAAAACTCG	1617
	R: TATCCGTGTAGATGTTGACG	
REV-F5	F: AGCAGAATTGATCGCACTAA	1777
	R: ACAAGGCAGTCCCCAAAGTTGCTG	
REV-F6	F: CTTGAACGGCTTCCTTCCAT	768
	R: CCCCCAAATGTTGTAC	

**Table 3:** Primers used for the rescue of rHB2023.

Fragment	Primer Sequence (5′→3′)	Length (bp)
REV-A	F: CATGCCTGCAGGTCGACGATTAATGTGGGAGGG	2684
	R: GAATGAGTCACTAG(G)CCCCTCCCTAGGTCC	
REV-B	F: CTTGTGGACCTAGGGAGGGG(C)CTAGTGACT	3078
	R: CCCGGGGATCCTCTAGAGATTGGACTTAAGCAAGGT	
REV-C	F: ACCTTGCTTAAGTCCCTACAGGCC	2627
	R: CCATGATTACGAATTCCCCCAAATGTTGTA	

Note: Underlined nucleotides indicate restriction sites, and those in parentheses denote designed genetic markers.

## Data Availability

The original contributions presented in this study are included in the article/Supplementary Material. Further inquiries can be directed to the corresponding author.

## Supplementary Materials

The following supporting information can be downloaded at: https://www.mdpi.com/article/10.3390/vetsci13101084/s1, Figure S1: Genome segmentation amplification results of the HB2301 strain; Figure S2: Nucleotide sequence identity matrices of REV HB2301 strain and representative REV isolates across different genome regions; Figure S3: Nucleotide and amino acid sequence alignment confirming the synonymous nature of the A2649C mutatione; Table S1: REV reference strain information; Table S2: Genomic regions of reticuloendotheliosis virus strain HB2301.
