# Supplementary material for: Isolation, Identification, Genomic Analysis, and Rescue of a Field-Derived Isolate of Reticuloendotheliosis Virus from Hubei, China
Source: Vet Sci. 2026 Oct 13;13(10):1084. doi: 10.3390/vetsci13101084 (PMC13709632; doi:10.3390/vetsci13101084)
Supplement: Supplementary file 1 [file vetsci-13-01084-s001.zip › supplementary material.pdf]

Figure S1. Genome segmentation amplification results of the HB2301 strain.

Lane M: DNA Marker AL2000; lanes F1 to F6 correspond to the amplified overlapping fragments F1, F5, F2, F3, F4, and F6.

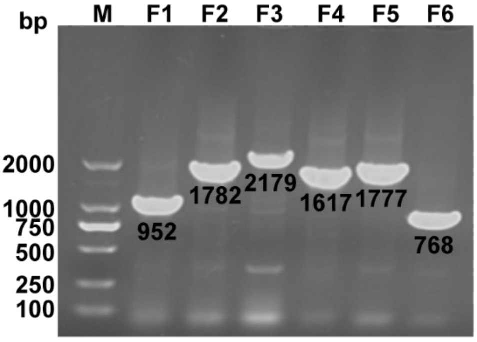

Figure S2. Nucleotide sequence identity matrices of REV HB2301 strain and representative REV isolates across different genome regions.(A) Complete genome; (B) Long terminal repeat (LTR) region; (C) *gag* gene; (D) *pol* gene. The matrices display the pairwise percent identity (upper right) and divergence (lower left) values calculated using MegAlign (DNASTAR). Darker or higher values indicate greater sequence identity. Strain details including accession numbers, subtypes, host species, sampling locations, and collection years are provided in Supplementary Table S1.

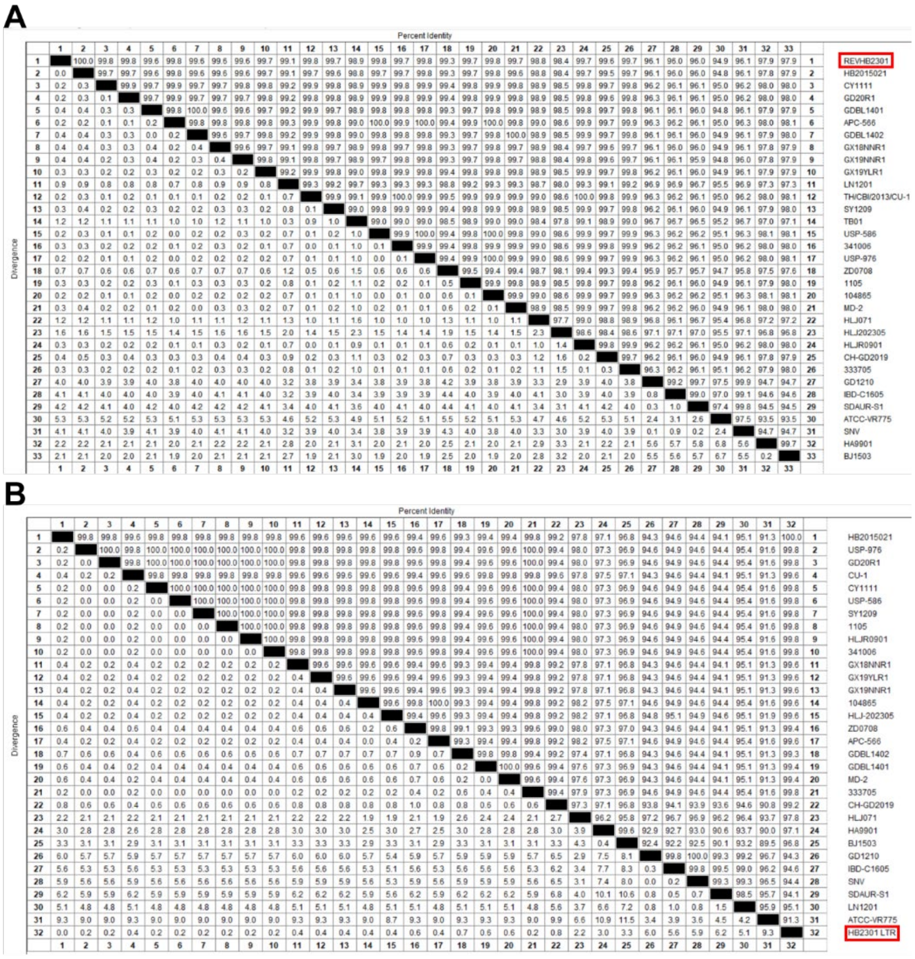

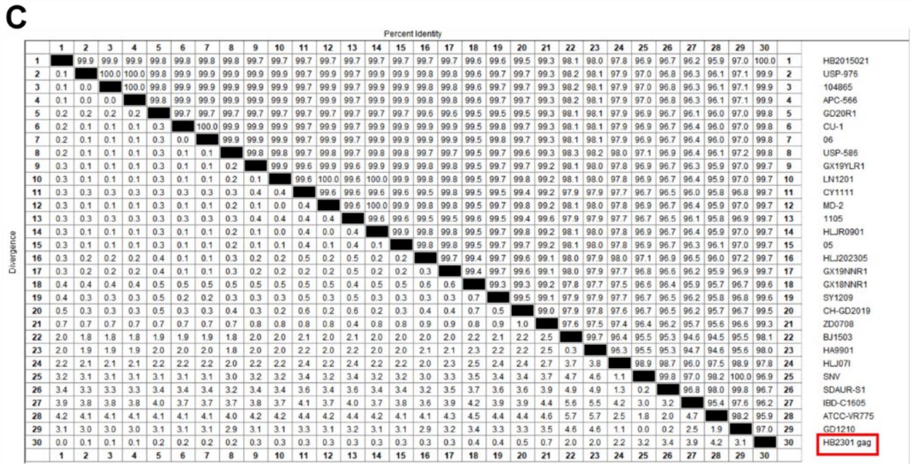

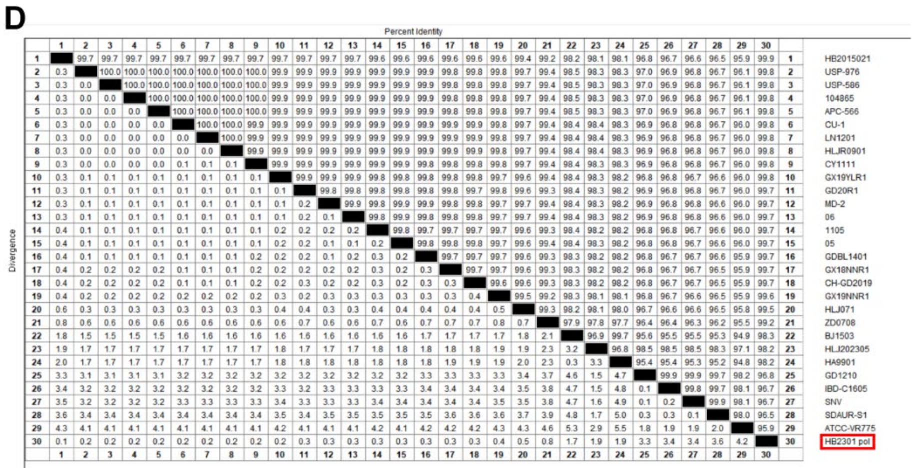

Figure S3. Nucleotide and amino acid sequence alignment confirming the synonymous nature of the A2649C mutation. Alignment of the partial nucleotide and deduced amino acid sequences of the wild-type HB2301 (top) and the recombinant virus rHB2301 (bottom). The A-to-C substitution at genomic position 2649 is highlighted in red (C). Both the wild-type codon GGA and the mutant codon GGC encode the same amino acid, glycine (Gly). The translated amino acid sequences (Gly-Arg-Gly-Leu) are identical between the two viruses, confirming that A2649C is a synonymous mutation.

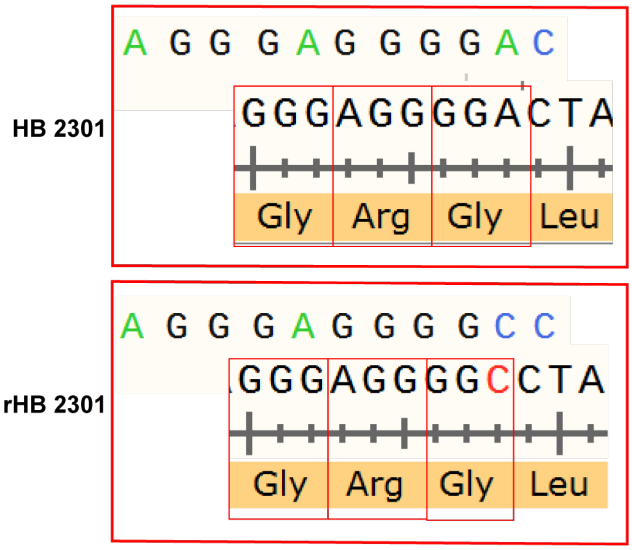

Table S1. REV reference strain information

Strain name	Accession no.	Subtype	Source	Yr	Host
REV-T	GU222419	I	USA	1957	Chicken
HA9901	NC006934.1	I	China	1999	Chicken
FPV	AF246698	III	USA	2003	FPV
3337-05	FJ439120	III	Taiwan, China	2005	Chicken
3410-06	FJ439119	III	Taiwan, China	2006	Goose
ZD0708	FJ496333	III	China	2007	Chicken
MD-2	JX912710	III	China	2008	MDV vaccine
HLJR0901	GQ415646	III	China	2009	Chicken
170A	GU222420	I	USA	2010	Chicken
DBYR1101	KC884559	III	China	2011	Anas acuta
DBYR1102	KC884555	III	China	2011	Anas platyrhynchos
DBYR1103	KC884562	III	China	2011	Anas formosa
DBYR1106	KC884561	III	China	2011	Anas poecilorhyncha
DBYR1201	KC884554	III	China	2012	Aythya fuligula

HLJ071	GQ375848.1	III	China	2012	chicken
LN1201	KU641115	III	China	2012	Chicken
SY1209	KJ909530	III	China	2012	Chicken
1105	JQ804915	III	China	2012	Duck
DBYR1203	KC884557	III	China	2012	Tarsiger cyanurus
GD1210	KF709431	II	China	2012	Chicken
TH/CBI/2013/CU-1	MF631845	III	Thailand	2013	Chicken
ATCC-VR775	KF313137.1	II	USA	2013	Duck
104865	KJ756349.1	III	USA	2014	turkey
GDBL1402	KU204703	III	China	2014	Pigeon
GDBL1401	KU204702	III	China	2014	Pigeon
CY1111	KJ909531	III	China	2015	Chicken
HB2015021	KY581581.1	III	China	2015	chicken
BJ1503	MG471384.1	I	China	2015	Chicken
APC-566	DQ387450.1	III	USA	2016	Attwater's prairie chickens
SNV	DQ003591.1	II	USA	2016	Chicken
IBD-C1605	KX278301	II	China	2016	IBDV vaccine
SDAUR-S1	MF185397	II	China	2017	Chicken
GX18NNR1	MW046297	III	China	2018	Chicken
GX19YLR1	MW046296	III	China	2019	Chicken
GX19NNR1	MW046295	III	China	2019	Chicken
CH-GD2019	MN812764	III	China	2019	Duck
GD20R1	MW046294	III	China	2020	Chicken
TB01	MZ833509	III	China	2021	Chicken
USP-586	OK631657.1	III	Brazil	2022	Gallus gallus
USP-976	OK631658.1	III	Brazil	2022	Gallus gallus
HLJ202305	PV361571.1	III	China	2023	Chicken

Table S2. Genomic regions of reticuloendotheliosis virus strain HB2301

Genomic region	Length（bp）	Position in genome (nt)
5’LTR	543	1-543
gag	1500	934-2433
pol	3582	2434-6015
env	1761	5952-7712
3’LTR	544	7742-8285
